# Stimulatory effects of activated water combined with foliar iron fertilization on photosynthetic characteristics and yield of pakchoi

**DOI:** 10.3389/fpls.2025.1671786

**Published:** 2025-10-02

**Authors:** Yan Sun, Tiantian Wang, Mingyue Li, Yichen Wang, Quanjiu Wang

**Affiliations:** ^1^ State Key Laboratory of Water Engineering Ecology and Environment in Arid Area, Xi’an University of Technology, Xi’an, China; ^2^ Xinjiang Future Irrigation District Engineering Technology Research Center, Urumqi, China

**Keywords:** activated water irrigation, foliar iron fertilizer, photosynthetic light-response, modified rectangularhyperbola model, pakchoi yield

## Abstract

The scarcity of freshwater resources, coupled with declining land productivity caused by imbalances in macro- and micronutrient ratios, severely constrains agricultural output and threatens sustainable development. At the same time, evolving dietary patterns and rising living standards have substantially increased vegetable consumption, making vegetables the second-largest crop in China after staple food crops. Therefore, this study investigated a coupled treatment system combining activated water irrigation with iron fertilization. The objectives were to conserve water resources, optimize nutrient allocation, enhance vegetable yield and quality, and provide a theoretical basis and technical support for promoting the sustainable development of the vegetable industry. Pakchoi (Brassica rapa subsp. chinensis) was selected as the experimental crop and subjected to three irrigation methods (tap water, magnetized water, and de-electronized water) under five iron fertilizer concentrations (0, 12.5, 25, 37.5, and 50 mg/L). Photosynthetic responses and yield characteristics were analyzed. Four light-response curve models (rectangular hyperbola, modified rectangular hyperbola, non-rectangular hyperbola, and exponential models) were applied to fit the photosynthetic light-response curves. Results showed that the M-Fe-1/2 treatment achieved the highest net photosynthetic rate (*P_n_*). The maximum net photosynthetic rate (*P_n_*_max_) initially increased and then decreased with rising iron concentration, peaking at 25 mg/L. Model evaluation indicated that the modified rectangular hyperbola model provided the best fit, with the coefficient of determination (*R²*) closest to 1 and the lowest root mean square error (RMSE) and mean absolute error (MAE), establishing it as the optimal model for simulating light-response curves under activated water and iron co-application. Under magnetized water irrigation combined with 25 mg/L iron, model parameters—including *P_n_*_max_, apparent quantum efficiency (*α*), dark respiration rate (*R_d_*), light saturation point (*I_sat_*), and light compensation point (*I_c_*)—all reached optimal levels, while shoot fresh weight increased significantly (38.21%–100.37% higher than other treatments). These findings demonstrate that magnetized water irrigation combined with 25 mg/L iron fertilizer markedly enhances pakchoi photosynthetic capacity, activates photosynthetic metabolism, promotes assimilate accumulation, and ultimately increases yield.

## 1 Introduction

With societal advancement and a rapidly growing population, water demand has intensified dramatically (Qu et al., 2020). Consequently, improving water conservation and utilization efficiency has become a critical issue. Activated irrigation water technology, an emerging approach, provides novel perspectives for enhancing the physiological potential of irrigation water and improving its comprehensive efficiency in agricultural ecosystems (Quanjiu et al., 2019). This technology modifies the physicochemical properties of ordinary water—including pH, electrical conductivity, surface tension, viscosity, and permeability—through treatments such as magnetization, de-electronization, and oxygenation, thereby enhancing molecular activity (Mu et al., 2019; Maheshwari and Grewal, 2009). Magnetized water is generated when water flows through a magnetic field at a certain velocity, cutting perpendicularly across magnetic induction lines. This process alters the structure of water molecules, accelerates their movement, and improves absorption efficiency by crops, ultimately contributing to water-saving effects (Zhao et al., 2022). De-electronized water is produced by removing negatively charged electrons carried by water molecules, causing mutual repulsion and separation. This reduces surface tension and alters hydrogen bonding associations, enabling more water molecules to infiltrate fine soil pores during percolation and improving soil water-holding capacity (Wei et al., 2021). Such treatments not only enhance soil physicochemical properties, promote crop growth and yield, and improve water use efficiency, but also remain simple, low-energy, cost-effective, pollution-free, and efficient (Zhao et al., 2021). For these reasons, activated irrigation water technology has received considerable attention and holds promise as a strategy to alleviate water scarcity.

Photosynthesis, the fundamental process by which plants convert solar energy into chemical energy (Anbarasan and Ramesh, 2022), is a central determinant of growth and development. The efficiency with which crops capture light and convert carbon dioxide and water into essential biomolecules ultimately governs yield potential (Ma et al., 2020; Simkin et al., 2019; Hu et al., 2020). Photosynthetic efficiency is regulated by environmental factors such as light intensity, temperature, and water availability, with micronutrient bioavailability serving as a critical limitation. Micronutrients are essential for enzymatic reactions and electron transport chains, ensuring both structural integrity and functional maintenance of the photosynthetic apparatus (Yamori et al., 2022; Kolbert et al., 2022). Among these, iron is the most abundant essential micronutrient in plants, playing key roles in photosynthesis, respiration, and chlorophyll biosynthesis (Kobayashi and Nishizawa, 2012). Approximately 80%–90% of cellular iron is localized in chloroplasts, where it maintains electron transfer between photosystems; insufficient iron leads to photooxidative damage (Kobayashi and Nishizawa, 2012). Deficiency also impairs nutrient uptake and physiological activity, causing chlorosis (Merry et al., 2022). Research has shown that foliar iron supplementation enhances photosynthetic pigment content (Zhang R. et al., 2022; Okla et al., 2023) and net photosynthetic rate (Zhang et al., 2023), whereas deficiency induces interveinal chlorosis through structural damage to leaf cells (Xing et al., 2010). In contrast, excessive foliar iron application diminishes pigment content, triggers lipid membrane peroxidation, and ultimately impairs plant growth through pigment degradation (Gao et al., 2022).

Foliar fertilization, an integral component of modern high-yield agriculture, enables precise nutrient delivery throughout all growth stages (Januszkiewicz et al., 2023). This targeted, rapid, and environmentally friendly technique has gained widespread attention for improving crop productivity under both optimal and stress conditions. It is widely employed to correct micronutrient deficiencies during critical developmental phases, alleviate soil nutrient limitations, and enhance nutrient use efficiency (Ishfaq et al., 2022). Because soil-applied micronutrients are often immobilized and rendered unavailable for uptake, foliar application serves as an effective alternative to increase micronutrient utilization (Bana et al., 2022; Hussein et al., 2024). Previous studies have demonstrated that foliar iron fertilization significantly increases photosynthetic pigment content, particularly chlorophyll, enhances photosynthetic efficiency, and improves fruit quality traits (Zhang et al., 2023). In addition, activated water irrigation stimulates physiological activity by enhancing electron transfer efficiency in redox systems and promoting iron translocation within plants, thereby improving both photosynthetic performance and shoot biomass (Sun et al., 2024). De-electronated water, characterized by its lower surface tension and smaller water molecular clusters, enhances water permeability and mobility within the soil-plant system, thereby improving water and nutrient uptake efficiency. Simultaneously, it may help regulate cellular redox homeostasis, potentially reducing reactive oxygen species accumulation and protecting the photosynthetic apparatus from photo-oxidative damage.Zheng et al. (2024) further reported that magneto-electrical water irrigation combined with iron fertilization resulted in higher iron accumulation than conventional tap water irrigation, as the reduced surface tension of magneto-electrical water improves droplet wettability and adhesion on leaf surfaces, thereby increasing spray efficacy.

Light-response models have become increasingly important for simulating photosynthetic light-response curves, which describe the relationship between net photosynthetic rate and photosynthetic photon flux density. These models are valuable tools for evaluating photosynthetic activity, light-use capacity, and plant adaptation mechanisms, as well as for assessing primary productivity (Ye et al., 2013; Kieffer et al., 2024). Key models include the rectangular hyperbola, non-rectangular hyperbola, exponential, and modified rectangular hyperbola, which generate core photosynthetic parameters such as maximum net photosynthetic rate, apparent quantum efficiency, dark respiration rate, light compensation point, and light saturation point. These parameters are critical for evaluating crop photosynthesis, light-use efficiency, and related physiological processes that influence growth and productivity (Guo and Lv, 2025). For example, Zhou et al. (2022) showed that suitable light intensity improves lettuce photosynthetic capacity and yield under different temperature conditions. Similarly, Sun et al. (2023) demonstrated through comparative analyses of photosynthetic parameters—including apparent quantum efficiency, light saturation point, and light compensation point—that magneto-electrical water irrigation enhances stomatal regulation in spinach under water stress, broadens the effective light intensity range, and maintains normal photosynthetic performance. Pakchoi (Brassica rapa subsp. chinensis), a highly efficient leafy vegetable rich in minerals and vitamins, is characterized by a short growth cycle and a high multiple cropping index (Wang T. et al., 2022). However, with the intensification of cropping systems, iron deficiency has emerged as a major constraint on pakchoi yield (Wang Y. et al., 2022). Current research has largely focused on activated water irrigation in combination with macronutrients, while studies investigating its interaction with micronutrients in vegetable production remain limited. Therefore, this study analyzes pakchoi’s photosynthetic light-response characteristics under magnetized and de-electronized water irrigation, using four light-response models (rectangular hyperbola, non-rectangular hyperbola, exponential, and modified rectangular hyperbola) for curve-fitting analysis to identify the most suitable model and its characteristic parameters. The results provide theoretical foundations and methodological support for sustainable pakchoi production and efficient cultivation management. The main innovation of this study lies in developing and validating a precision fertilization strategy that enables synergistic regulation of macronutrients and micronutrients (Zn). By establishing a dynamic ratio model based on crop physiological requirements, this approach addresses the limitations of conventional methods that rely on a single nutrient supply.

## 2 Materials and methods

### 2.1 Experimental design

The pot experiment was conducted in the intelligence climate chamber of the State Key Laboratory of Water Engineering Ecology and Environment in Arid Area at Xi’an University of Technology, under controlled conditions of 25°C temperature, 50% humidity, and 40 Klux light intensity. The tested soil samples were collected from the 0–20 cm surface layer in Yangling, Shaanxi Province, with a soil type classified as medium loam. The measured available iron content in the test soil sample was 2.35 mg/kg. The activated water (magnetized water and de-electronated water) used in the experiment was prepared in the laboratory. De-electronated water was prepared by passing tap water through a de-electronation device at a constant flow rate. This device exported electrons from the tap water via an externally connected grounding resistor. Magnetized water was prepared by passing tap water through a permanent magnet (manufactured by Baotou Xinda Magnetic Materials Factory) with a magnetic field strength of 3000 Gs at a constant flow rate (0.05 m/s,10s), resulting in water magnetization through the applied magnetic field.

The experiment was divided into three groups, irrigated with tap water, magnetized water, and de-electronated water, respectively. The tap water irrigation treatment served as the control (CK). Each group included five iron application gradients, resulting in a total of 15 treatments (**Table 1**) with three replicates per treatment.

**Table 1:** Experimental design treatments.

Fe application treatment (mg⋅L^-1^)	Irrigation water		
	CK	E	M
0	CK-Fe-0	E-Fe-0	M-Fe-0
12.5	CK-Fe-1/4	E-Fe-1/4	M-Fe-1/4
25	CK-Fe-1/2	E-Fe-1/2	M-Fe-1/2
37.5	CK-Fe-3/4	E-Fe-3/4	M-Fe-3/4
50	CK-Fe-1	E-Fe-1	M-Fe-1

CK is tap water, M is magnetized water, and E is de-electronated water; 0, 1/4, 1/2, 3/4, and 1 correspond to multiples of the full iron fertilizer application rate (50 mg/L), specifically 0, 12.5, 25, 37.5, and 50 mg/L, respectively.

The potted soil was filled according to a bulk density of 1.3 g/cm^3^. The pots had an upper diameter of 14 cm, a lower diameter of 9 cm, and a height of 15 cm. Urea (100 mg/kg), calcium phosphate (50 mg/kg), and potassium sulfate (50 mg/kg) were added according to conventional application rates for potted pakchoi. The pots were irrigated to field capacity with tap water, magnetized water, or de-electronated water, and then placed in a closed greenhouse for 24-hour equilibration before sowing.

After 24 hours of soil equilibration, plump pakchoi seeds were selected and uniformly sown at 20 seeds per pot on the soil surface. The seeds were covered with 50 g of dry soil and watered with 10 mL of water. Seven days after sowing, the seedlings were thinned to 10 plants per pot. Iron was then applied as ferrous sulfate heptahydrate (FeSO_4_·7H_2_O) at iron concentrations of 0, 1/4, 1/2, 3/4, and 1 times the 50 mg/L rate (Sun et al., 2024). The iron solutions were thoroughly mixed and uniformly sprayed onto the upper and lower surfaces of pakchoi leaves using a small manual sprayer. The application volume was adjusted to form droplets on the leaves without runoff. Spraying was repeated every 10 days for a total of three applications.

### 2.2 Measurement methods

The light response curves of pakchoi during its vigorous vegetative growth stage were measured using an LC-pro portable photosynthesis system. Measurements were conducted on sunny days between 09:00-11:00, with three healthy plants randomly selected per treatment (fully expanded upper leaves). Each leaf had three replicate readings (averaged). The instrument’s built-in red-blue light source was used to measure net photosynthetic rates (*P_n_*) under different photosynthetically active radiation (PAR) levels. Fourteen PAR gradients (2000, 1600, 1400, 1200, 1000, 800, 600, 400, 200, 100, 80, 50, 20, and 0 *µ*mol/(m^2^⋅s)) were applied in descending order to account for light induction requirements. The curves yielded key photosynthetic parameters: apparent quantum efficiency(*α*), maximum net photosynthetic rate (*P_n_*_max_), dark respiration rate (*R_d_*), light saturation point (*I_sat_*), and light compensation point (*I_c_*). At maturity, all plants were harvested, and fresh shoot biomass was determined with three biological replicates per plant.

### 2.3 Light-response curve models

Four different models were employed to analyze the light response curves of pakchoi, with their mathematical formulations specified as follows:

(1) the rectangular hyperbola model (Thornley, 1976) is expressed as:


(1) $$P_{n}=\frac{\alpha IP_{n\max}}{\alpha I+P_{n\max}}-R_{d}$$

In the equation, *P_n_* is net photosynthetic rate (*µ*mol/(m^2^⋅s)), *α* is apparent quantum efficiency, *P_n_*_max_ is maximum net photosynthetic rate (*µ*mol/(m^2^⋅s)), *R_d_* is dark respiration rate (*µ*mol/(m^2^⋅s)), and I is photosynthetically active radiation (*µ*mol/(m^2^⋅s)).

The light compensation point can be calculated using the following equation:


(2) $$I_{c}=\frac{R_{d}P_{n\max}}{\alpha (P_{n\max}-R_{d})}$$

The light saturation point is determined by the x-axis value at the intersection of the straight line y = *P_n_*_max_ and the line y =*αI*-*R_d_*.

(2) Modified rectangular hyperbola model (Ye, 2007) is expressed as:


(3) $$P_{n}=\alpha \frac{1-\beta I}{1+\gamma I}(I-I_{c})$$

In the equation, *I*_c_ is the light compensation point, *β* is the photoinhibition coefficient, and *γ* is a coefficient independent of *I*.

The dark respiration rate is defined as:


(4) $$R_{d}=-P(I=O)=-\alpha I_{c}$$

The light saturation point is defined as:


(5) $$I_{sat}=\frac{\sqrt{(\beta +\gamma )(1+\gamma I_{c})/\beta }-1}{\gamma }$$

The maximum net photosynthetic rate is defined as:


(6) $$P_{n\max}=\alpha \frac{1-\beta I_{sat}}{1+\gamma I_{sat}}(I_{sat}-I_{c})$$

The quantum efficiency at *I* = 0 is defined as the intrinsic quantum efficiency(*Φ_0_*):


(7) $$\Phi_{0}=P'(I=0)=\alpha [1+(\gamma +\beta )I_{c}]$$

The quantum efficiency at *I*=*I_c_* (*Φ_c_*) (Aspinwall et al., 2011) is defined as:


(8) $$\Phi_{c}=P'(I=I_{c})=\alpha \frac{1+(\gamma -\beta )I_{c}-\beta \gamma I_{c}^{2}}{{\left(1+\gamma I_{c}\right)}^{2}}$$

In the equation, *Φ_c_* represents the apparent quantum efficiency of plants at the light compensation point.

The absolute value of the slope (*Φ_c0_*) connecting the points at *I* = 0 and *I*=*I_c_* on the light-response curve is defined as:


(9) $$\Phi_{c0}=|P(I=0)/I_{c}|=\alpha$$

(3) non-rectangular hyperbola model (Thornley, 1976) is expressed as:


(10) $$P_{n}=\frac{\alpha I+P_{n\max}-\sqrt{{\left(\alpha I+P_{n\max}\right)}^{2}-4I\alpha kP_{n\max}}}{2k}-R_{d}$$

In the equation, k is the curvature parameter of the non-rectangular hyperbola model.

If the model demonstrates satisfactory fitting, the light compensation point (*I_c_*) can be calculated using the following equation:


(11) $$I_{c}=\frac{R_{d}P_{n\max}-kR_{d}^{2}}{\alpha (P_{n\max}-R_{d})}$$

The light saturation point (*I_sat_*) is determined by the x-coordinate of the intersection between the horizontal line y = *P_n_*_max_ and the linear function y=*αI*-*R_d_*.

(4) Exponential models (Thornley, 1976) is mathematically expressed as:


(12) $$P_{n}=P_{n\max}(1-e^{-aI/P_{n\max}})-R_{d}$$

For *I_sat_* estimation, the light saturation point was operationally defined as the photosynthetically active radiation (PAR) corresponding to 0.99*P_n_*_max_ (where *P_n_* reaches 99% of its maximum value).

The light saturation point (*I_sat_*) cannot be precisely determined from rectangular hyperbola, non-rectangular hyperbola or exponential models directly, since these are all monotonically increasing functions. Instead, *I*_sat_ was calculated by first fitting the light response data measured under low light intensity (≤200 *µ*mol/(m^2^⋅s)) with a linear equation to obtain the apparent quantum efficiency (*α*), and then solving the equation *P_n_*_max_ =*αI*-*R_d_*.

### 2.4 Data processing

Data were organized using Excel 2019. Statistical analyses were performed with SPSS 27.0 software. All data were first subjected to normality tests (Shapiro-Wilk test) and homogeneity of variance tests (Levene’s test). After verifying that the data met the assumptions for parametric tests, one-way analysis of variance (ANOVA) was employed to compare differences among treatments. If the ANOVA results indicated significant differences (*p* < 0.05), the least significant difference (LSD) method was further employed for *post-hoc* multiple comparisons. Data visualization was conducted using Origin 2021 software. Model fitting accuracy was evaluated through three indicators: root mean square error (RMSE), mean absolute error (MAE), and coefficient of determination (*R* ^2^), calculated as follows:


(13) $$R^{2}=1-\frac{\sum_{i=1}^{n}{\left(O_{i}-S_{i}\right)}^{2}}{\sum_{i=1}^{n}{\left(O_{i}-\bar{O_{i}}\right)}^{2}}$$

(14) $$RMSE=\sqrt{\frac{\sum_{i=1}^{n}{\left(O_{i}-S_{i}\right)}^{2}}{n}}$$

(15) $$MAE=\frac{1}{n}\sum_{i=1}^{n}\left|O_{i}-S_{i}\right|$$

In the equations, *O_i_* is observed value, *S_i_* is simulated value, $\bar{O_{i}}$ is mean of observed values, and n is sample size.

## 3 Results and analysis

### 3.1 Determination of optimal light-response curve model

Under activated water irrigation combined with foliar iron application, the net photosynthetic rate (*P_n_*) of pakchoi initially increased and then decreased with rising photosynthetically active radiation (PAR). The modified rectangular hyperbola model accurately captured this unimodal response pattern, whereas the rectangular hyperbola (Equations 1, 2), non-rectangular hyperbola (Equations 10, 11), and exponential models (Equation 12) generated monotonically increasing functions. Thus, the modified rectangular hyperbola model better represented the light response characteristics of pakchoi leaves, enabling more precise estimation of key photosynthetic parameters: apparent quantum efficiency (*α*), maximum net photosynthetic rate (*P_n_*_max_), light compensation point (*I_c_*), light saturation point (*I_sat_*), and dark respiration rate (*R_d_*). Comparative analysis of the four models (**Table 2**) revealed that for activated water irrigation with iron fertilization, the modified rectangular hyperbola model exhibited superior performance - its coefficient of determination (*R^2^*, Equation 13) was closer to 1, while showing lower root mean square error (RMSE, Equation 14) and mean absolute error (MAE, Equation 15) values compared to other models. These results confirm that the modified rectangular hyperbola model is the optimal choice for fitting light response curves of pakchoi under activated water irrigation with iron fertilization.

**Table 2:** Comparative analysis of simulation accuracy among four light-response models under different coupled treatments of activated water irrigation and iron fertilizer application.

Fe gradient	Model	RMSE			MAE			R^2^		
		CK	E	M	CK	E	M	CK	E	M
Fe-0	Rectangular hyperbola model	1.617	0.494	1.339	1.258	0.427	0.856	0.960	0.988	0.928
	Modified rectangular hyperbola model	0.344	0.341	0.492	0.281	0.270	0.431	0.998	0.994	0.990
	Non-rectangular hyperbola model	1.107	0.405	0.606	0.975	0.354	0.521	0.978	0.997	0.943
	Exponential model	1.207	0.301	1.169	0.831	0.254	0.662	0.978	0.996	0.945
Fe-1/4	Rectangular hyperbola model	1.704	0.323	1.354	1.262	0.253	1.052	0.921	0.997	0.955
	Modified rectangular hyperbola model	0.327	0.154	0.118	0.290	0.131	0.092	0.997	0.999	0.999
	Non-rectangular hyperbola model	0.896	0.261	0.939	0.756	0.227	0.776	0.948	0.999	0.980
	Exponential model	1.359	0.258	0.899	0.855	0.216	0.607	0.950	0.998	0.980
Fe-1/2	Rectangular hyperbola model	1.197	1.251	1.012	0.891	0.966	0.792	0.973	0.973	0.993
	Modified rectangular hyperbola model	0.469	0.336	0.314	0.411	0.274	0.229	0.996	0.998	0.999
	Non-rectangular hyperbola model	0.775	0.933	0.834	0.681	0.833	0.751	0.982	0.988	0.998
	Exponential model	0.884	0.833	0.494	0.647	0.632	0.310	0.986	0.988	0.998
Fe-3/4	Rectangular hyperbola model	0.823	0.474	0.721	0.712	0.375	0.477	0.964	0.992	0.995
	Modified rectangular hyperbola model	0.204	0.155	0.497	0.173	0.133	0.438	0.998	0.999	0.998
	Non-rectangular hyperbola model	0.625	0.372	0.273	0.529	0.329	0.237	0.988	0.997	0.996
	Exponential model	0.439	0.261	0.598	0.329	0.222	0.497	0.989	0.998	0.997
Fe-1	Rectangular hyperbola model	0.865	1.524	1.508	0.603	1.116	1.061	0.978	0.930	0.979
	Modified rectangular hyperbola model	0.349	0.294	0.631	0.284	0.245	0.513	0.996	0.997	0.998
	Non-rectangular hyperbola model	0.492	0.920	0.944	0.418	0.762	0.844	0.985	0.956	0.987
	Exponential model	0.628	1.165	1.092	0.379	0.715	0.652	0.988	0.959	0.989

CK is tap water, E is de-electronated water, M is magnetized water; 0, 1/4, 1/2, 3/4, and 1 correspond to multiples of the full iron fertilizer application rate (50 mg/L), specifically 0, 12.5, 25, 37.5, and 50 mg/L, respectively. RMSE is root mean square error, MAE is mean absolute error, and R^2^ is determination coefficient.

### 3.2 Characteristics of light-response curve parameters

Key photosynthetic parameters of pakchoi leaves under activated water irrigation coupled with iron fertilization, including apparent quantum efficiency (*α*), maximum net photosynthetic rate (*P_n_*_max_), light compensation point (*I_c_*), light saturation point (*I_sat_*), and dark respiration rate (*R_d_*), were calculated using the modified rectangular hyperbola model (**Table 3**, Equations 3-9). The 95% confidence intervals for photosynthetic characteristic parameters are shown in **Table 4**.

**Table 3:** Photosynthetic light-response parameters of pakchoi leaves under coupled treatments of activated water irrigation and iron fertilization.

Irrigation water	Fe gradient	*α*	*P*_nmax_ (*µ*mol⋅m^-2^⋅s^-1^)	*I_c_* (*µ*mol⋅m^-2^⋅s^-1^)	*I_sat_* (*µ*mol⋅m^-2^⋅s^-1^)	*R_d_* (*µ*mol⋅m^-2^⋅s^-1^)	*△I*=*I_sat -_ I_c_* (*µ*mol⋅m^-2^⋅s^-1^)
CK	Fe-0	0.077 ± 0.003a	9.527 ± 0.262d	45.090 ± 2.562b	1208.992 ± 10.289b	-3.459 ± 0.253a	1163.902 ± 12.850c
	Fe-1/4	0.059 ± 0.003b	14.158 ± 0.170bc	51.973 ± 2.124a	1791.424 ± 18.248a	-3.067 ± 0.072a	1739.452 ± 20.290a
	Fe-1/2	0.052 ± 0.002c	18.265 ± 0.198a	33.811 ± 1.122c	1213.102 ± 21.461b	-1.762 ± 0.207b	1179.291 ± 22.565bc
	Fe-3/4	0.080 ± 0.002a	13.441 ± 0.298c	16.908 ± 1.146d	1248.432 ± 26.537b	-1.355 ± 0.184b	1231.523 ± 25.583b
	Fe-1	0.078 ± 0.002a	14.994 ± 0.498b	19.427 ± 1.089d	862.162 ± 20.170c	-1.516 ± 0.178b	842.735 ± 19.221d
E	Fe-0	0.051 ± 0.002c	20.251 ± 0.200a	17.404 ± 0.940bc	1166.261 ± 20.560b	-0.895 ± 0.189b	1148.857 ± 21.212a
	Fe-1/4	0.053 ± 0.001c	15.651 ± 0.174c	18.505 ± 0.953b	977.788 ± 16.174d	-0.974 ± 0.137b	959.283 ± 16.996c
	Fe-1/2	0.048 ± 0.002c	17.609 ± 0.371b	31.211 ± 1.174a	1226.143 ± 14.940a	-1.508 ± 0.276b	1194.933 ± 16.050a
	Fe-3/4	0.085 ± 0.002a	9.867 ± 0.579d	35.617 ± 1.557a	843.184 ± 15.423e	-3.012 ± 0.510a	807.567 ± 14.161d
	Fe-1	0.069 ± 0.001b	14.830 ± 0.269c	12.892 ± 1.209c	1087.827 ± 24.784c	-0.892 ± 0.119b	1074.935 ± 25.563b
M	Fe-0	0.022 ± 0.002e	12.945 ± 0.278d	6.190 ± 1.610c	1227.708 ± 16.972c	-0.138 ± 0.113c	1221.518 ± 15.629c
	Fe-1/4	0.070 ± 0.002b	16.594 ± 0.202c	15.220 ± 2.414b	985.789 ± 21.978d	-1.072 ± 0.063b	970.569 ± 19.690d
	Fe-1/2	0.081 ± 0.002a	30.199 ± 0.348a	23.779 ± 1.405a	1541.342 ± 23.356b	-1.924 ± 0.011a	1517.563 ± 22.051b
	Fe-3/4	0.054 ± 0.002d	26.054 ± 0.293b	15.048 ± 1.404b	1807.607 ± 20.914a	-0.813 ± 0.066b	1792.558 ± 22.171a
	Fe-1	0.063 ± 0.002c	25.627 ± 0.225b	16.297 ± 2.030b	1278.654 ± 16.775c	-1.033 ± 0.285b	1262.357 ± 18.496c

CK is tap water, E is de-electronated water, M is magnetized water; 0, 1/4, 1/2, 3/4, and 1 correspond to multiples of the full iron fertilizer application rate (50 mg/L), specifically 0, 12.5, 25, 37.5, and 50 mg/L, respectively. *α* is apparent quantum efficiency, *P_n_*_max_ is maximum net photosynthetic rate, *I_c_* is light compensation point, *I_sat_* is light saturation point, *R_d_* is dark respiration rate, *ΔI* is light adaptation range.

**Table 4:** The 95% confidence intervals for photosynthetic parameters of pakchoi under different irrigation water types and iron fertilizer levels.

Irrigation water	Fe gradient	*α*		*P*_nmax_		*I_c_*		*I_sat_*		*R_d_*		*△I*	
		Lower limit	Upper limit	Lower limit	Upper limit	Lower limit	Upper limit	Lower limit	Upper limit	Lower limit	Upper limit	Lower limit	Upper limit
CK	Fe-0	0.075	0.079	9.159	9.895	43.204	46.976	1185.796	1232.188	-3.711	-3.207	1140.493	1187.311
	Fe-1/4	0.057	0.061	13.790	14.526	50.087	53.859	1768.228	1814.62	-3.319	-2.815	1716.043	1762.861
	Fe-1/2	0.050	0.054	17.897	18.633	31.925	35.697	1189.906	1236.298	-2.014	-1.510	1155.882	1202.700
	Fe-3/4	0.078	0.082	13.073	13.809	15.022	18.794	1225.236	1271.628	-1.607	-1.103	1208.114	1254.932
	Fe-1	0.076	0.080	14.626	15.362	17.541	21.313	838.966	885.358	-1.768	-1.264	819.326	866.144
E	Fe-0	0.049	0.053	19.883	20.619	15.518	19.290	1143.065	1189.457	-1.147	-0.643	1125.448	1172.266
	Fe-1/4	0.051	0.055	15.283	16.019	16.619	20.391	954.592	1000.984	-1.226	-0.722	935.874	982.692
	Fe-1/2	0.046	0.050	17.241	17.977	29.325	33.097	1202.947	1249.339	-1.760	-1.256	1171.524	982.692
	Fe-3/4	0.083	0.087	9.499	10.235	33.731	37.503	819.988	866.380	-3.264	-2.760	784.158	830.976
	Fe-1	0.067	0.071	14.462	15.198	11.006	14.778	1064.631	1111.023	-1.144	-0.640	1051.526	1098.344
M	Fe-0	0.020	0.024	12.577	13.313	4.304	8.076	1204.512	1250.904	-0.390	-0.114	1198.109	1244.927
	Fe-1/4	0.068	0.072	16.226	16.962	13.334	17.106	962.593	1008.985	-1.324	-0.820	947.160	993.978
	Fe-1/2	0.079	0.083	29.831	30.567	21.893	25.665	1518.146	1564.538	-2.176	-1.672	1494.154	1540.972
	Fe-3/4	0.052	0.056	25.686	26.422	13.162	16.934	1784.411	1830.803	-1.065	-0.561	1769.149	1815.967
	Fe-1	0.061	0.065	25.259	25.995	14.411	18.183	1255.458	1301.85	-1.285	-0.781	1238.948	1285.766

CK is tap water, E is de-electronated water, M is magnetized water; 0, 1/4, 1/2, 3/4, and 1 correspond to multiples of the full iron fertilizer application rate (50 mg/L), specifically 0, 12.5, 25, 37.5, and 50 mg/L, respectively.

Under tap water irrigation (CK), the apparent quantum efficiency (α) in CK-Fe-0, CK-Fe-3/4, and CK-Fe-1 treatments was significantly higher than in CK-Fe-1/4 and CK-Fe-1/2 (*p* < 0.05), indicating greater light energy absorption under low irradiance. Specifically, CK-Fe-3/4 increased α by 35.59% and 53.85% compared with CK-Fe-1/4 and CK-Fe-1/2, respectively (*p* < 0.05). With de-electronated water irrigation (E), the E-Fe-3/4 treatment exhibited significantly higher α than E-Fe-0, E-Fe-1/4, E-Fe-1/2, and E-Fe-1 (*p* < 0.05), with increases of 66.67%, 58.49%, 77.08%, and 23.19%, respectively. This indicates that E-Fe-3/4 achieves optimal photosynthetic efficiency under low light. Under magnetized water irrigation (M), the M-Fe-1/2 treatment showed the highest *α*, surpassing M-Fe-0, M-Fe-1/4, M-Fe-3/4, and M-Fe-1 by 268.18%, 15.71%, 50.00%, and 28.57%, respectively (*p* < 0.05). These results confirm that M-Fe-1/2 maximizes light energy conversion efficiency under low-irradiance conditions.

Under CK irrigation, the CK-Fe-1/2 treatment exhibited the highest maximum net photosynthetic rate (*P_n_*_max_), exceeding CK-Fe-0, CK-Fe-1/4, CK-Fe-3/4, and CK-Fe-1 by 91.72%, 29.01%, 35.89%, and 21.82%, respectively (*p* < 0.05). This demonstrates that CK-Fe-1/2 most effectively enhances light-use efficiency under conventional irrigation. With de-electronated water irrigation (E), the E-Fe-0 treatment produced the highest *P_n_*_max_, surpassing E-Fe-1/4, E-Fe-1/2, E-Fe-3/4, and E-Fe-1 by 29.39%, 15.00%, 105.24%, and 36.55%, respectively (*p* < 0.05). This suggests that E-Fe-0 promotes superior light absorption capacity in the absence of iron supplementation. For magnetized water irrigation (M), the M-Fe-1/2 treatment achieved peak photosynthetic performance, with *P_n_*_max_ values 133.29%, 81.99%, 15.91%, and 17.84% higher than M-Fe-0, M-Fe-1/4, M-Fe-3/4, and M-Fe-1, respectively (*p* < 0.05). These findings confirm that magnetized water combined with 25 mg/L Fe (M-Fe-1/2) maximizes photosynthetic capacity.

Under CK irrigation, the CK-Fe-3/4 and CK-Fe-1 treatments exhibited significantly lower light compensation points (*I_c_*) than CK-Fe-0, CK-Fe-1/4, and CK-Fe-1/2 (*p* < 0.05), with reductions of 62.50%, 67.46%, 49.99%, 56.92%, 62.62%, and 42.54%, respectively. These results indicate improved low-light utilization efficiency, making CK-Fe-3/4 and CK-Fe-1 more suitable for shaded environments. With de-electronated water irrigation (E), the E-Fe-1 treatment recorded the lowest *I_c_*, decreasing by 25.93%, 30.33%, 58.69%, and 63.80% compared with E-Fe-0, E-Fe-1/4, E-Fe-1/2, and E-Fe-3/4, respectively (*p* < 0.05). This suggests that E-Fe-1 maximizes light absorption under low irradiance. Under magnetized water irrigation (M), the M-Fe-0 treatment demonstrated the lowest *I_c_*, with reductions of 59.33%, 73.97%, 58.86%, and 62.02% relative to M-Fe-1/4, M-Fe-1/2, M-Fe-3/4, and M-Fe-1 (*p* < 0.05). This highlights the superior low-light adaptability of pakchoi in the absence of iron fertilization under magnetized water conditions.

Under CK irrigation, the CK-Fe-1/4 treatment exhibited the highest light saturation point (*I_sat_*), exceeding CK-Fe-0, CK-Fe-1/2, CK-Fe-3/4, and CK-Fe-1 by 48.18%, 47.67%, 43.49%, and 107.78%, respectively (*p* < 0.05). This indicates that CK-Fe-1/4 improves tolerance to strong light, making it suitable for high-irradiance conditions. With de-electronated water irrigation (E), the E-Fe-1/2 treatment showed the highest *I_sat_*, surpassing E-Fe-0, E-Fe-1/4, E-Fe-3/4, and E-Fe-1 by 5.13%, 25.40%, 45.42%, and 12.71%, respectively (*p* < 0.05). These results demonstrate superior adaptation to intense light under E-Fe-1/2. For magnetized water irrigation (M), the M-Fe-3/4 treatment achieved the highest *I_sat_*, showing increases of 47.23%, 83.37%, 17.27%, and 41.37% over M-Fe-0, M-Fe-1/4, M-Fe-1/2, and M-Fe-1, respectively (*p* < 0.05). This indicates that M-Fe-3/4 maximizes photosynthetic performance under strong light.

Under CK irrigation, the CK-Fe-1/4 treatment exhibited the broadest light adaptation range (*ΔI* = *I_sat_* – *I_c_*), exceeding CK-Fe-0, CK-Fe-1/2, CK-Fe-3/4, and CK-Fe-1 by 49.45%, 47.50%, 41.24%, and 106.41%, respectively (*p* < 0.05). This demonstrates that CK-Fe-1/4 substantially enhances adaptability across variable irradiance levels. With de-electronated water irrigation (E), both E-Fe-0 and E-Fe-1/2 showed superior *ΔI* values, exceeding E-Fe-1/4, E-Fe-3/4, and E-Fe-1 by 19.76%, 42.26%, and 6.88% (E-Fe-0) and by 24.57%, 47.97%, and 11.16% (E-Fe-1/2), respectively (*p* < 0.05). These results indicate efficient light utilization under both low and high irradiance conditions. For magnetized water irrigation (M), the M-Fe-3/4 treatment achieved the broadest *ΔI*, with increases of 46.75%, 84.69%, 18.12%, and 42.00% compared with M-Fe-0, M-Fe-1/4, M-Fe-1/2, and M-Fe-1, respectively (*p* < 0.05), confirming its superior adaptability to fluctuating light intensities.

Under CK irrigation, the CK-Fe-0 and CK-Fe-1/4 treatments exhibited significantly higher dark respiration rates (*R_d_*) than CK-Fe-1/2, CK-Fe-3/4, and CK-Fe-1 (*p* < 0.05), with increases of 96.31%, 155.28%, and 128.17% (CK-Fe-0) and 74.06%, 126.35%, and 102.31% (CK-Fe-1/4), respectively. These findings suggest elevated physiological activity in these treatments, likely reflecting greater metabolic demand. With de-electronated water irrigation (E), the E-Fe-3/4 treatment displayed the highest *R_d_*, exceeding E-Fe-0, E-Fe-1/4, E-Fe-1/2, and E-Fe-1 by 236.54%, 209.24%, 99.73%, and 237.67%, respectively (*p* < 0.05). This indicates peak metabolic activity under E-Fe-3/4, potentially associated with active growth processes. Under magnetized water irrigation (M), the M-Fe-1/2 treatment exhibited the highest *R_d_* compared with M-Fe-0, M-Fe-1/4, M-Fe-3/4, and M-Fe-1 (*p* < 0.05), suggesting the strongest physiological activity and the highest rate of organic matter consumption in pakchoi under this treatment.

### 3.3 Effect sizes (partial η^2^) of photosynthetic parameters under water and fertilizer treatments

Irrigation water type, iron fertilizer rate, and their interaction significantly affected all photosynthetic parameters (*p* < 0.001), with substantial effect sizes (**Table 5**). The interaction between irrigation water and iron fertilization demonstrated the highest effect sizes (0.945≤partial η^2 ≤^ 0.995), identifying it as the predominant factor governing the photosynthetic physiological characteristics of pakchoi. Notably, this interaction accounted for more than 99.5% of the variance in light saturation point (*I_sat_*), light saturation range (*ΔI*), and maximum net photosynthetic rate (*P_n_*_max_), demonstrating pronounced biological effects of the experimental treatments. These results highlight that the synergistic water-fertilizer coupling effect plays a decisively greater role in determining photosynthetic performance than the independent contributions of either factor alone.

**Table 5:** Partial η^2^ values for the effects of irrigation water type, iron fertilizer gradient, and their interaction on photosynthetic parameters.

Source	*α*	*P*_nmax_	*I_c_*	*I_sat_*	*R_d_*	*△I*
Irrigation Water	0.886	0.995	0.970	0.984	0.895	0.985
Fe gradient	0.959	0.991	0.932	0.968	0.624	0.965
Irrigation Water*Fe gradient	0.984	0.995	0.978	0.995	0.945	0.995

All effects were statistically significant (*p* < 0.001). *α* represents apparent quantum efficiency, *P_n_*_max_ denotes maximum net photosynthetic rate, *I_c_* indicates light compensation point, *I_sat_* signifies light saturation point, *R_d_* stands for dark respiration rate, *ΔI* shows the range of available light intensity.

### 3.4 Characteristics of light-response curve variations

#### 3.4.1 Effects of foliar iron fertilization on pakchoi light-response curves under different activated water irrigation regimes

Under irrigation with tap water, de-electronated water, and magnetized water, the net photosynthetic rate (*P_n_*) of pakchoi showed a decelerating increase once photosynthetically active radiation (PAR) reached 400 μmol/(m²·s).

**Figure 1:**
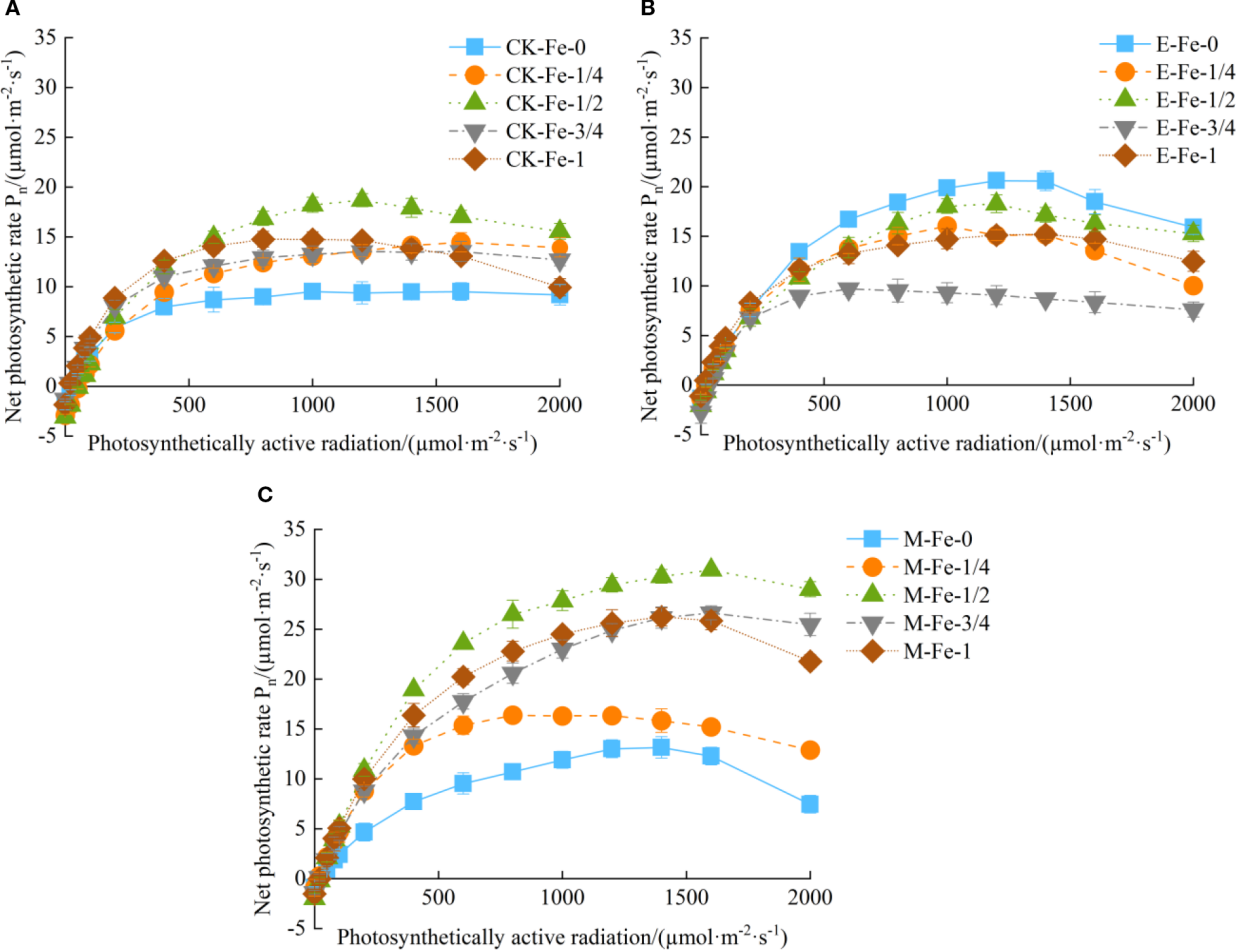
Effects of foliar iron fertilization on light-response curves of pakchoi under different activated water irrigation regimes. CK is tap water, E is de-electronated water, M is magnetized water; 0, 1/4, 1/2, 3/4, and 1 correspond to multiples of the full iron fertilizer application rate (50 mg/L), specifically 0, 12.5, 25, 37.5, and 50 mg/L, respectively. **(A–C)** represent light-response curves of CK, E, and M treatments, respectively. Among these, the M-Fe-1/2 treatment in **(C)** achieved the highest net photosynthetic rate.

Under CK irrigation, at 1200 µmol/(m²·s), *P_n_* in the CK-Fe-1/2, CK-Fe-3/4, and CK-Fe-1 treatments began to decline with further increases in PAR, whereas CK-Fe-1/4 and CK-Fe-0 showed only minimal reductions even at 1500 µmol/(m²·s) (**Figure 1A**). The maximum *P_n_* occurred at 1200 µmol/(m²·s), with CK-Fe-1/2 significantly exceeding CK-Fe-0, CK-Fe-1/4, CK-Fe-3/4, and CK-Fe-1 by 99.47%, 37.28%, 38.30%, and 27.44%, respectively.

Under de-electronated water irrigation (E), the E-Fe-3/4 treatment exhibited declining *P_n_* once PAR exceeded 600 µmol/(m²·s), while E-Fe-0, E-Fe-1/4, and E-Fe-1/2 showed decreases only after 1200 µmol/(m²·s). Notably, E-Fe-1 declined only at the highest PAR level of 1500 µmol/(m²·s) (**Figure 1B**). The maximum *P_n_* was achieved at 1200 µmol/(m²·s), with E-Fe-0 performing best—36.51%, 12.88%, 127.62%, and 36.33% higher than E-Fe-1/4, E-Fe-1/2, E-Fe-3/4, and E-Fe-1, respectively. This indicates that the absence of iron supplementation (E-Fe-0) under de-electronated water irrigation most effectively sustained photosynthetic activity across varying light intensities.

Under magnetized water irrigation (M), the M-Fe-1/4 treatment showed reduced *P_n_* above 800 µmol/(m²·s), whereas M-Fe-0, M-Fe-1/2, M-Fe-3/4, and M-Fe-1 maintained *P_n_* until 1600 µmol/(m²·s) before declining (**Figure 1C**). Peak photosynthetic performance was observed at 1600 µmol/(m²·s), with M-Fe-1/2 significantly surpassing M-Fe-0, M-Fe-1/4, M-Fe-3/4, and M-Fe-1 by 152.28%, 103.49%, 16.23%, and 19.70%, respectively. These results demonstrate that moderate iron supplementation (M-Fe-1/2) combined with magnetized water optimally enhances light tolerance and photosynthetic capacity in pakchoi.

#### 3.4.2 Effects of different activated water irrigation types on pakchoi light-response characteristics under foliar iron fertilization

**Figure 2:**
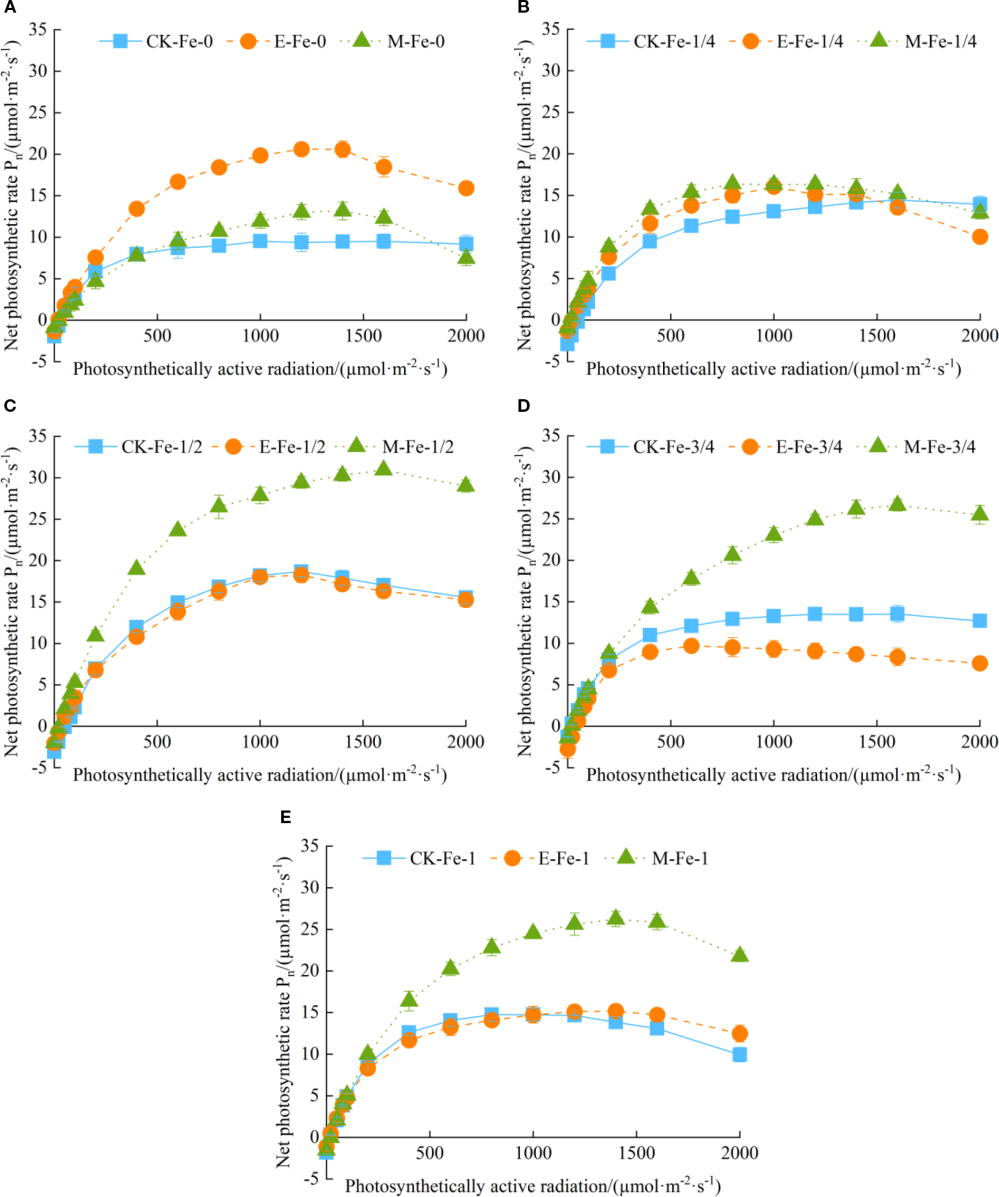
Effects of different activated water irrigation types on light-response characteristics of pakchoi leaves under foliar iron fertilization. CK is tap water, E is de-electronated water, M is magnetized water; 0, 1/4, 1/2, 3/4, and 1 correspond to multiples of the full iron fertilizer application rate (50 mg/L), specifically 0, 12.5, 25, 37.5, and 50 mg/L, respectively. **(A–E)** represent the light-response curves of Fe-0, Fe-1/4, Fe-1/2, Fe-3/4, and Fe-1 treatments, respectively. Among these, the M-Fe-1/2 treatment in **(C)** achieved the highest net photosynthetic rate.

Under the Fe-0 treatment, *P_n_* of pakchoi under CK-Fe-0, E-Fe-0, and M-Fe-0 exhibited a typical unimodal response to increasing PAR, with initial enhancement followed by gradual decline (**Figure 2A**). The light-response curves of CK-Fe-0 and M-Fe-0 showed statistically similar patterns. At 1200 µmol/(m²·s), E-Fe-0 reached a maximum *P_n_* of 20.60 µmol/(m²·s), representing increases of 120.09% and 58.46% compared with CK-Fe-0 and M-Fe-0, respectively.

Under the Fe-1/4 treatment, pakchoi plants in CK-Fe-1/4, E-Fe-1/4, and M-Fe-1/4 showed a typical response in which *P_n_* initially rose and then declined with increasing PAR (**Figure 2B**). The light-response curves of M-Fe-1/4 and E-Fe-1/4 were nearly identical. At 800 µmol/(m²·s), M-Fe-1/4 achieved peak photosynthetic performance of 16.38 µmol/(m²·s), 31.99% higher than CK-Fe-1/4 and 9.27% higher than E-Fe-1/4.

Under the Fe-1/2 treatment, pakchoi plants in CK-Fe-1/2, E-Fe-1/2, and M-Fe-1/2 displayed a consistent unimodal response, with *P_n_* first increasing and then decreasing as PAR rose (**Figure 2C**). The light-response curves of CK-Fe-1/2 and E-Fe-1/2 showed highly similar patterns. At 1600 µmol/(m²·s), M-Fe-1/2 achieved exceptional photosynthetic performance, with a maximum *P_n_* of 30.93 µmol/(m²·s). This represented increases of 81.62% over CK-Fe-1/2 and 89.64% over E-Fe-1/2, demonstrating the superior efficacy of magnetized water at this optimal iron level.

Under the Fe-3/4 treatment, all irrigation systems—CK-Fe-3/4, E-Fe-3/4, and M-Fe-3/4—exhibited unimodal responses, with *P_n_* rising initially and then declining due to photoinhibition (**Figure 2D**). CK-Fe-3/4 consistently maintained higher *P_n_* than E-Fe-3/4 across the PAR range. At 1600 µmol/(m²·s), M-Fe-3/4 achieved peak performance (26.61 µmol/(m²·s)), 96.67% higher than CK-Fe-3/4 and 219.45% higher than E-Fe-3/4.

Under the Fe-1 treatment, pakchoi plants in CK-Fe-1, E-Fe-1, and M-Fe-1 also showed unimodal *P_n_* responses to increasing PAR (**Figure 2E**). The light-response curves of CK-Fe-1 and E-Fe-1 were highly similar, with no significant differences. At 1400 µmol/(m²·s), M-Fe-1 achieved peak *P_n_* of 26.21 µmol/(m²·s), 89.52% higher than CK-Fe-1 and 72.66% higher than E-Fe-1.

In summary, foliar iron application did not significantly improve *P_n_* in pakchoi under de-electronated water irrigation. Under de-electronated water irrigation, the *P_n_* in the E-Fe-0 treatment was higher than in all iron-supplied treatments. This outcome may be attributed to photo-oxidative damage induced by iron addition. Consequently, in the absence of supplemental iron (E-Fe-0), the photosynthetic apparatus was better protected as photosynthetically active radiation increased. However, both tap water and magnetized water systems exhibited measurable gains with iron supplementation, with the most pronounced effect at the Fe-1/2 level. Among all treatments, M-Fe-1/2 achieved the highest overall *P_n_*, confirming this combination as the optimal protocol for maximizing photosynthetic efficiency. Comparative analysis further showed that magnetized water consistently outperformed tap water at 25, 37.5, and 50 mg/L Fe application rates.

### 3.5 Effects of activated water irrigation with iron fertilization on pakchoi shoot fresh weight

#### 3.5.1 Impact of foliar iron application on shoot fresh weight under different activated water irrigation regimes

Under CK, de-electronated water, and magnetized water irrigation treatments, the shoot fresh weight of pakchoi exhibited an increasing trend with the number of days after sowing. From days 14 to 21 after sowing, the differences in shoot fresh weight changes among the treatments were not significant.

**Figure 3:**
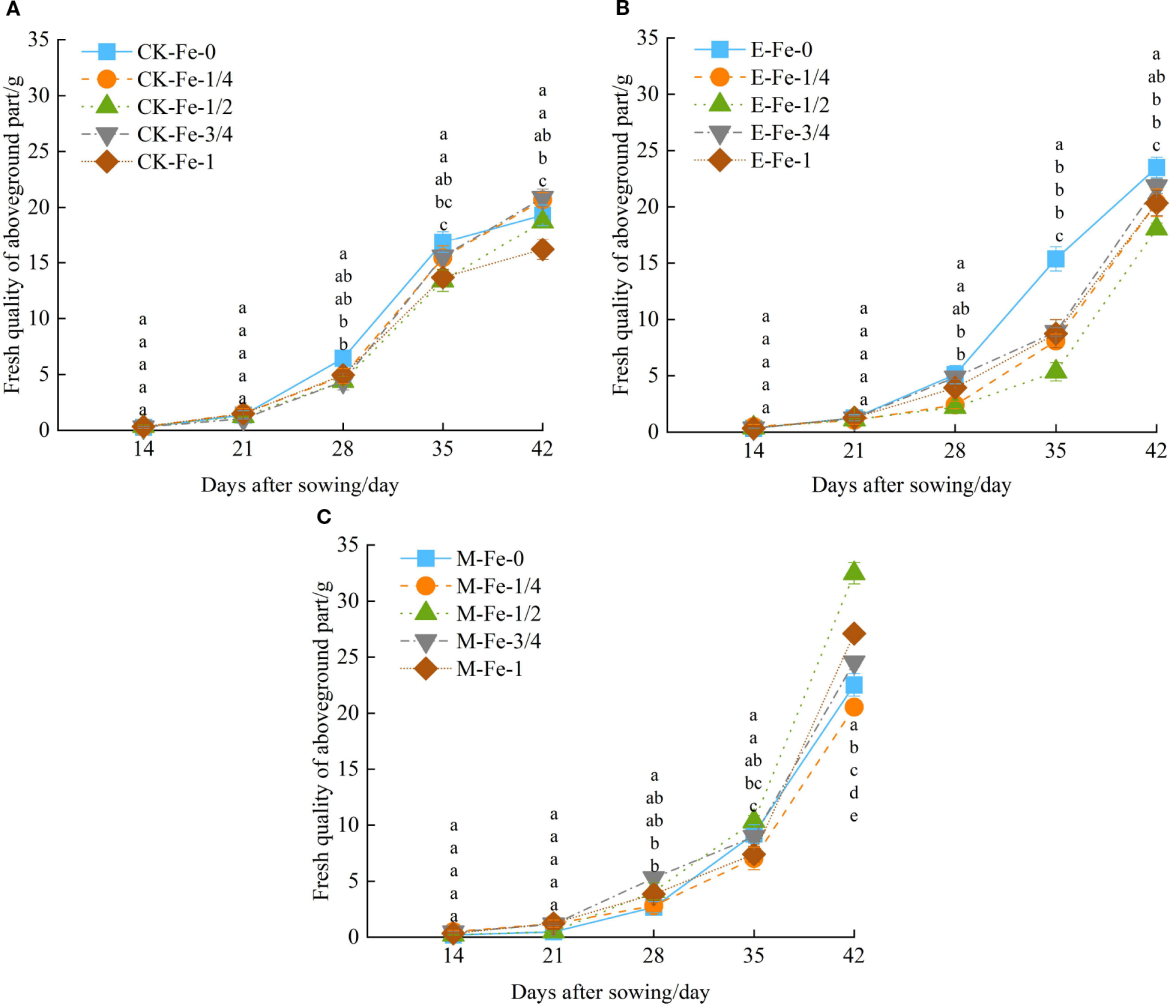
Effects of foliar iron fertilizer application on the fresh weight of pakchoi shoots under different activated water irrigation conditions. CK is tap water, E is de-electronated water, M is magnetized water; 0, 1/4, 1/2, 3/4, and 1 indicate multiples of the full iron fertilizer application rate (50 mg/L), corresponding to 0, 12.5, 25, 37.5, and 50 mg/L, respectively. Different lowercase letters indicate significant differences among treatments (*p* < 0.05). **(A–C)** represent the shoot fresh weight of pakchoi under CK, E, and M treatments, respectively. Among these, the M-Fe-1/2 treatment in **(C)** achieved the maximum fresh weight.

Under CK irrigation, from days 21 to 42, fresh weight growth followed a pattern of initial increase followed by decline (**Figure 3A**). At day 42, fresh weight under CK-Fe-1 was significantly lower than under CK-Fe-0, CK-Fe-1/4, CK-Fe-1/2, and CK-Fe-3/4, with reductions of 16.01%, 21.61%, 13.41%, and 22.35%, respectively (*p* < 0.05).

Under de-electronated water irrigation, from days 21 to 42, E-Fe-0 first increased and then declined, while E-Fe-1/4, E-Fe-1/2, E-Fe-3/4, and E-Fe-1 all showed continuous increases. From days 28 to 42, E-Fe-0 maintained consistently higher fresh weight than the other iron-supplied treatments (**Figure 3B**). At day 35, fresh weight under E-Fe-0 was significantly higher than under E-Fe-1/4, E-Fe-1/2, E-Fe-3/4, and E-Fe-1, by 90.11%, 187.65%, 72.94%, and 76.05%, respectively (*p* < 0.05). By day 42, E-Fe-0 still exceeded E-Fe-1/4, E-Fe-1/2, and E-Fe-1 by 15.40%, 30.14%, and 15.49%, respectively (*p* < 0.05). From days 35 to 42, fresh weight under E-Fe-1/2 was significantly lower than in other iron-supplied treatments (*p* < 0.05).

Under magnetized water irrigation,from days 14 to 42, growth continued to increase across treatments(**Figure 3C**). At day 42, M-Fe-1/2 produced significantly higher fresh weight than M-Fe-0, M-Fe-1/4, M-Fe-3/4, and M-Fe-1, with increases of 44.19%, 58.12%, 32.28%, and 19.82%, respectively (*p* < 0.05).

#### 3.5.2 Effects of different types of activated water irrigation on pakchoi shoot fresh weight under foliar iron fertilizer application

**Figure 4:**
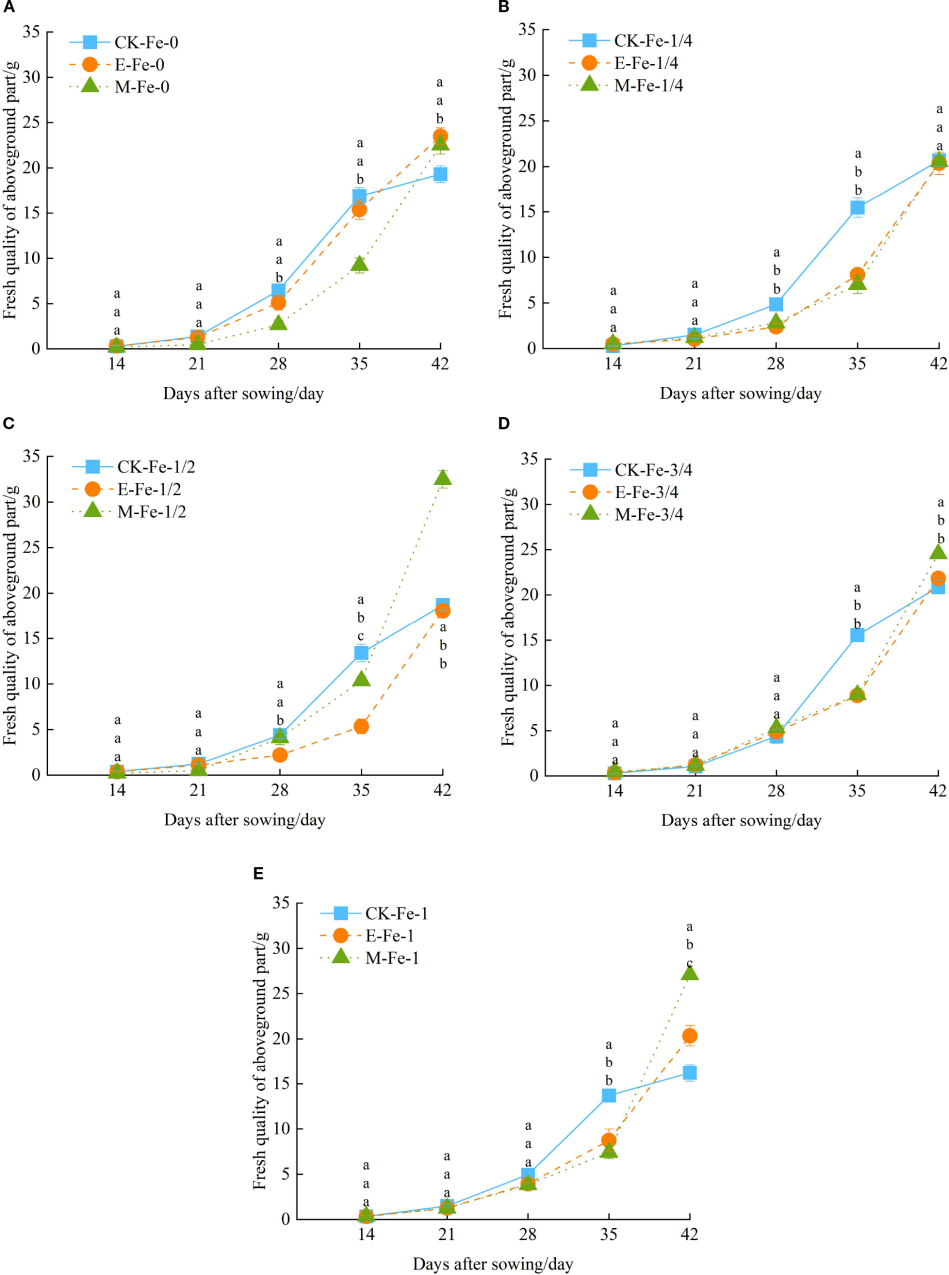
Effects of different activated water irrigation types on pakchoi shoot fresh weight under foliar iron fertilizer application. CK is tap water, E is de-electronated water, M is magnetized water; 0, 1/4, 1/2, 3/4, and 1 correspond to multiples of the full iron fertilizer application rate (50 mg/L), specifically 0, 12.5, 25, 37.5, and 50 mg/L, respectively. Different lowercase letters indicate statistically significant differences among treatments (*p* < 0.05). **(A–E)** represent the shoot fresh weight of pakchoi under Fe-0, Fe-1/4, Fe-1/2, Fe-3/4, and Fe-1 treatments, respectively. Among these, the M-Fe-1/2 treatment in **(C)** achieved the maximum fresh weight.

Under the Fe-0 treatment, the shoot fresh weight of pakchoi in all irrigation regimes showed an increasing trend with days after sowing. From day 14 to 21, no significant differences were observed among treatments. From day 14 to 35, all treatments exhibited similar patterns of shoot fresh weight accumulation. However, between day 35 and day 42, shoot fresh weight growth rates under E-Fe-0 and M-Fe-0 were significantly higher than under CK-Fe-0 (**Figure 4A**). At day 42, shoot fresh weights in E-Fe-0 and M-Fe-0 were 21.77% and 16.72% greater than CK-Fe-0, respectively (*p* < 0.05).

Under the Fe-1/4 treatment, shoot fresh weight in all irrigation systems increased steadily with cultivation time. From day 14 to 21, no significant differences were detected. From day 14 to 42, CK-Fe-1/4 displayed a growth pattern of initial acceleration followed by decline, whereas E-Fe-1/4 and M-Fe-1/4 maintained steady growth (**Figure 4B**). Notably, between day 28 and 35, shoot fresh weight under CK-Fe-1/4 was significantly higher than under both E-Fe-1/4 and M-Fe-1/4 (*p* < 0.05).

Under the Fe-1/2 treatment, all treatments exhibited progressive increases in shoot fresh weight throughout the cultivation period. During the early phase (14–21 days post-sowing), no significant differences were recorded among treatments. Between days 14 and 42, distinct patterns emerged: CK-Fe-1/2 followed a bell-shaped curve with growth acceleration followed by decline, while E-Fe-1/2 and M-Fe-1/2 maintained consistent growth acceleration (**Figure 4C**). At day 42, M-Fe-1/2 achieved markedly higher biomass than CK-Fe-1/2 and E-Fe-1/2, with increases of 73.50% and 79.87%, respectively (*p* < 0.05). This highlights the pronounced growth-promoting effect of magnetized water under moderate iron supplementation.

Under the Fe-3/4 treatment, all groups exhibited steady increases in shoot fresh weight during the 42-day growth period. In the early stage (14–28 days post-sowing), no significant differences were observed. Across the full growth cycle, CK-Fe-3/4 followed a curve peaking at day 35 and subsequently declining, whereas E-Fe-3/4 and M-Fe-3/4 maintained sustained growth (**Figure 4D**). At day 35, CK-Fe-3/4 significantly exceeded E-Fe-3/4 and M-Fe-3/4 by 74.99% and 73.41%, respectively (*p* < 0.05). However, by day 42 this trend reversed, with M-Fe-3/4 producing the highest fresh weight, exceeding CK-Fe-3/4 and E-Fe-3/4 by 17.62% and 12.44%, respectively (*p* < 0.05). This indicates a time-dependent advantage of magnetized water under high iron supplementation.

Under the Fe-1 treatment, all treatments exhibited progressive increases in shoot biomass throughout the 42-day period. During the initial phase (14–28 days after sowing, DAS), no significant differences were observed. From 14 to 42 DAS, distinct growth kinetics appeared: CK-Fe-1 displayed a unimodal curve, peaking near 28 DAS and subsequently declining, while E-Fe-1 and M-Fe-1 maintained sustained growth (**Figure 4E**). At 42 DAS, M-Fe-1 produced significantly higher shoot fresh weight than CK-Fe-1 and E-Fe-1, with increases of 67.22% and 33.21%, respectively (*p* < 0.05).

In summary, under CK irrigation, increasing iron application did not significantly improve pakchoi fresh weight. In contrast, both de-electronated and magnetized water enhanced fresh weight to varying degrees. Under magnetized water, shoot fresh weight generally followed a pattern of initial increase followed by decline with rising iron application rates. Among all treatments, magnetized water with 25 mg/L iron fertilizer (M-Fe-1/2) produced the strongest effect, increasing fresh weight by 68.30% compared with CK-Fe-0.

## 4 Discussion

This study confirmed that M-Fe-1/2 most effectively enhanced pakchoi leaf photosynthesis, with *P_n_* increases of 133.29%, 81.99%, 15.91%, and 17.84% relative to M-Fe-0, M-Fe-1/4, M-Fe-3/4, and M-Fe-1, respectively. These findings align with Sun et al. (2024), who demonstrated the synergistic effects of activated water and foliar iron fertilization in improving net photosynthetic rates. Magnetized irrigation water promotes cell division and growth, enhances shoot development, increases chlorophyll content, and improves leaf photosynthetic efficiency (Coley, 1983). Magnetization also improves water and nutrient uptake and increases mitochondrial abundance (Shabrangy and Ahmad, 2009; Nyakane et al., 2019), thereby providing additional sites for respiration and redox reactions, boosting energy supply, and enhancing growth parameters to increase yield. In contrast, under magnetized water irrigation, higher iron application rates significantly reduced *P_n_* (**Figure 1C**). This reduction was likely caused by the detrimental effects of excessive iron on chloroplast structural integrity, which disrupts chlorophyll biosynthesis and decreases the activity of photosynthesis-related enzymes, thereby impairing overall photosynthetic performance. Previous studies have shown that iron is directly involved in key biological processes, including photosynthesis and respiration, as it is an essential component for chlorophyll synthesis and plays a critical role in maintaining chlorophyll structure and function (El-Desouky et al., 2021; Nasar et al., 2022). These findings are consistent with Gao et al. (2022), who reported that excessive iron concentrations decrease pigment content and inhibit plant growth. Further evidence supports the beneficial role of magnetized water in crop production. Maheshwari and Grewal (2009) demonstrated that irrigation with magnetized water improved yields of celery and snow peas, while Putti et al. (2023) reported enhanced agronomic traits in lettuce, including leaf number, fresh and dry shoot weight, and root biomass. Additionally, Sun et al. (2024) showed that foliar iron fertilization increased total nitrogen accumulation in spinach leaves, enhanced chlorophyll content, and elevated the maximum net photosynthetic rate. Therefore, integrating activated water with an optimal iron concentration represents an effective strategy to enhance the physiological activity of irrigation water, improve photosynthetic efficiency, and promote sustainable crop productivity.

**Figure 5:**
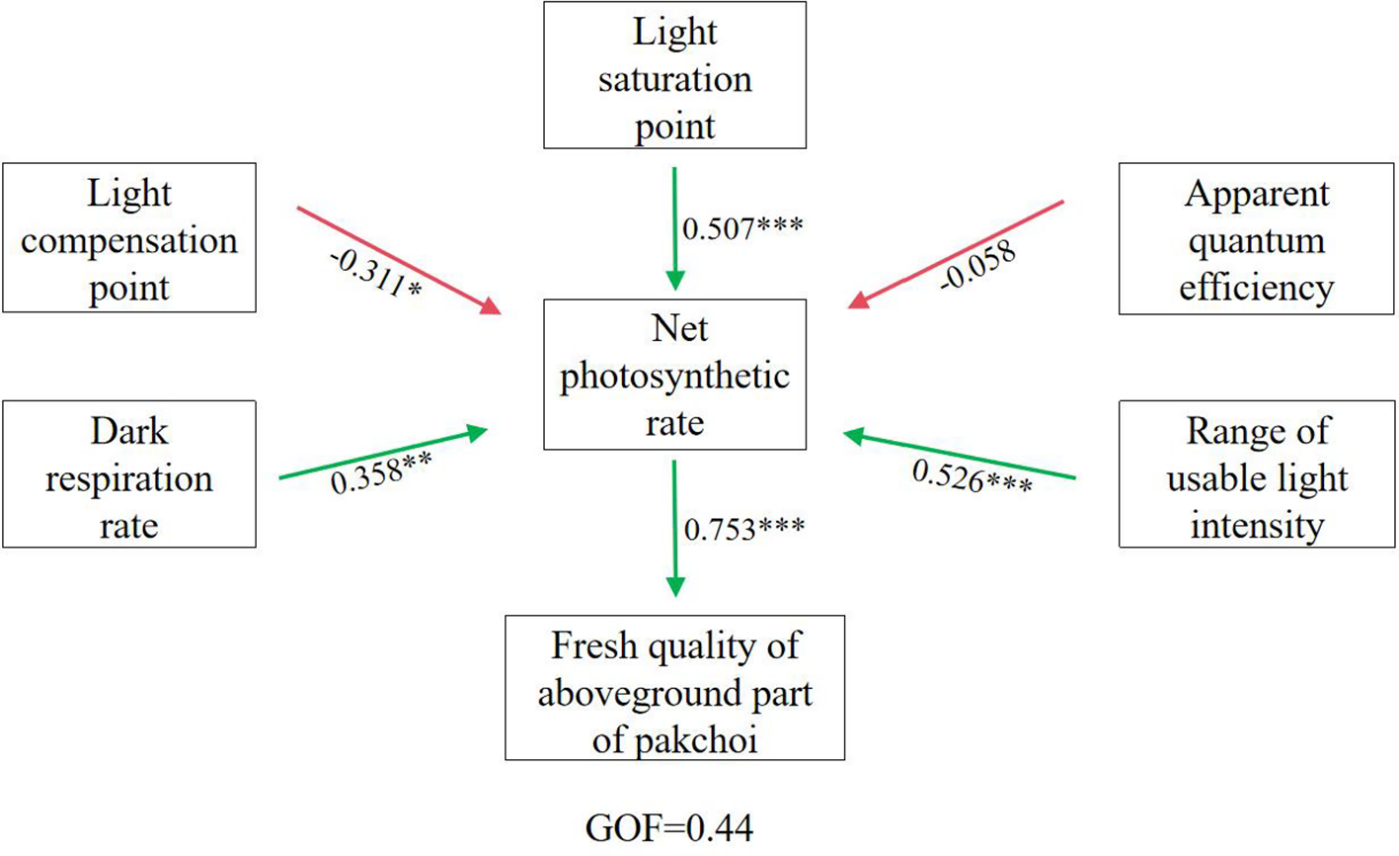
Structural equation modeling analysis results of different indicators in pakchoi. *, **, and *** indicate significant correlations at p < 0.05, p < 0.01, and p < 0.001 levels, respectively.

Photosynthetic characteristic parameters—apparent quantum efficiency, net photosynthetic rate, light compensation point, light saturation point, and dark respiration rate—are essential for evaluating crop photosynthesis, light-use efficiency, and related physiological processes influencing growth and productivity (Guo and Lv, 2025). The SEM results (**Figure 5**) showed that the influence coefficients of apparent quantum efficiency, light compensation point, light saturation point, dark respiration rate, and light intensity range on net photosynthetic rate were -0.058, -0.311, 0.507, 0.358, and 0.526, respectively. The net photosynthetic rate represents material accumulation during photosynthesis minus respiratory consumption, serving as a fundamental indicator of photosynthetic capacity (Zhang J. et al., 2022). Apparent quantum efficiency reflects the ability of crops to absorb and convert light under low-light conditions (Wan et al., 2018). In this study, apparent quantum efficiency showed a negative effect on *P_n_* (**Figure 5**), suggesting that strong light induced photoinhibition, leading to a concurrent reduction in both parameters. The light saturation point indicates the capacity of plants to utilize high irradiance, with higher values reflecting stronger adaptation to strong light environments (Sun et al., 2022). In this study, the light saturation point exerted a highly significant positive effect on *P_n_* (**Figure 5**), indicating that higher saturation points allow plants to better exploit high light intensities and sustain greater photosynthetic rates. The light compensation point corresponds to the irradiance level at which organic matter production equals respiratory consumption; lower values indicate more efficient use of weak light. Here, the light compensation point had a significant negative effect on *P_n_* (**Figure 5**), demonstrating that lower compensation points facilitate stronger photosynthetic activity under shaded conditions. Plants combining low light compensation points with high light saturation points possess greater adaptability to fluctuating light environments, whereas plants with high compensation points and low saturation points are less adaptable (Wan et al., 2018). To capture this adaptability, *ΔI* was used to represent the effective range of utilizable light intensity in pakchoi leaves. *ΔI* showed a highly significant positive effect on *P_n_* (**Figure 5**), suggesting that broader light adaptability enables plants to maintain stable photosynthetic performance across variable light environments. Dark respiration rate refers to plant respiration rate in darkness, with most released energy dissipated as heat and a small portion used for physiological activities. Dark respiration rate represents the rate of respiration in darkness, where most released energy dissipates as heat and a smaller portion supports metabolic activity. It reflects the physiological activity of leaves under dark conditions, with higher rates indicating greater nocturnal activity (Zhang et al., 2024). In this study, dark respiration rate exhibited a significant positive effect on *P_n_* (**Figure 5**), suggesting that moderate respiratory metabolism enhances nighttime physiological function and indirectly supports photosynthetic capacity.

**Figure 6:**
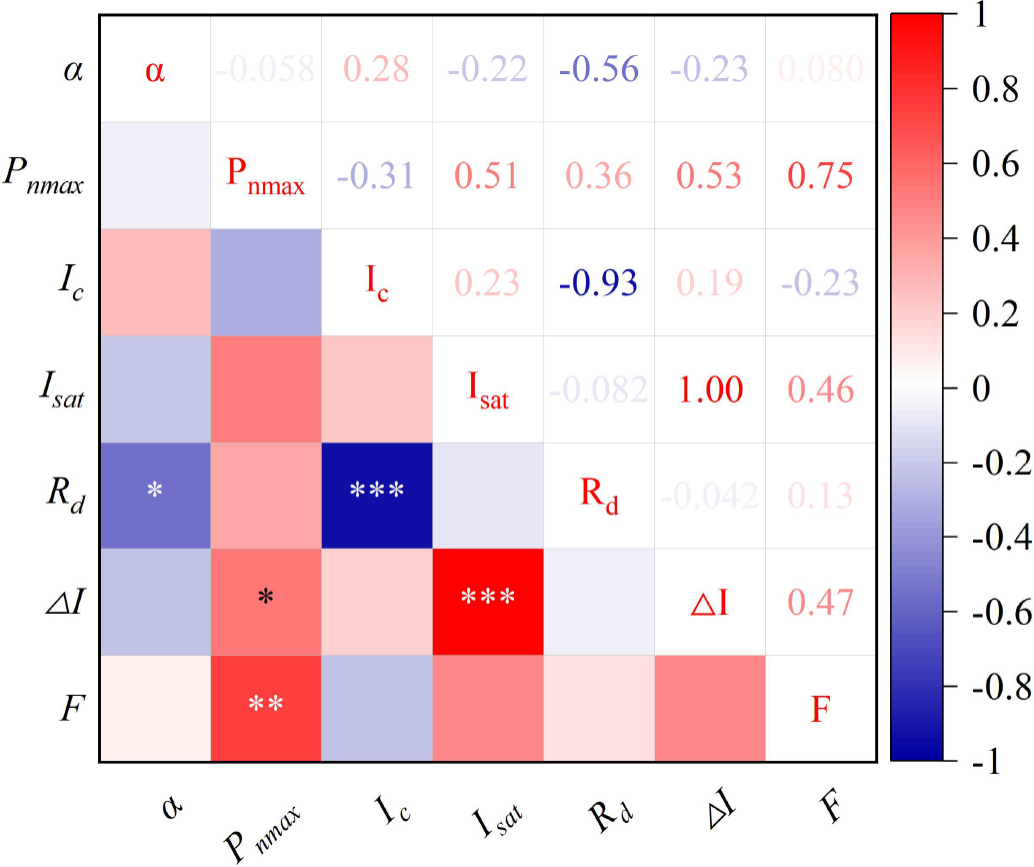
Correlation analysis of different indicators in pakchoi. *α* represents apparent quantum efficiency, *P_n_*_max_ denotes maximum net photosynthetic rate, *I_c_* indicates light compensation point, *I_sat_* signifies light saturation point, *R_d_* stands for dark respiration rate, *ΔI* shows the range of available light intensity, and *F* represents shoot fresh weight of pakchoi; *, **, and *** indicate significant correlations at p < 0.05, p < 0.01, and p < 0.001 levels, respectively.

Under magnetized water irrigation, the growth rate of pakchoi fresh weight first increased and then decreased with rising iron application levels (**Figure 3C**). The combined treatment of magnetized water with 25 mg/L iron produced the strongest effect, resulting in yield increases of 38.21%–100.37% compared with other treatments. These results are consistent with Caliskan et al. (2008), who reported that foliar iron application enhances crop yield. Similarly, Zhang R. et al. (2022) demonstrated that foliar iron fertilization improves both yield and quality, with increases ranging from 18.85% to 112.64%, likely due to improvements in photosynthetic pigment content, gas exchange parameters, and chlorophyll fluorescence. Structural equation modeling (**Figure 5**) further indicated that *P_n_* had a highly significant positive effect on pakchoi shoot fresh weight, demonstrating that higher *P_n_* supports greater carbohydrate synthesis, thereby providing energy for growth and promoting yield formation. The maximum net photosynthetic rate, which reflects the leaf’s light energy absorption capacity at saturation, represents an important indicator of photosynthetic potential (Niu et al., 2023). Correlation analysis (**Figure 6**) similarly revealed a strong positive relationship (coefficient = 0.75) between *P_n_* and shoot fresh weight, with all parameters except apparent quantum efficiency and light compensation point showing positive correlations with *P_n_*. Guiamba et al. (2022) also reported that enhanced photosynthesis positively influences yield through regulation of organ development, nutrient transport efficiency, and metabolic homeostasis. Collectively, these results confirm that *P_n_* is a key determinant of pakchoi yield formation.

In summary, photosynthetic parameters—including apparent quantum efficiency, net photosynthetic rate, light compensation point, light saturation point, dark respiration rate, and light intensity range—jointly regulate photosynthetic carbon assimilation efficiency. By promoting carbohydrate synthesis and accumulation, they significantly increase net photosynthetic rate. As a core physiological indicator, *P_n_* provides the material basis for crop growth by improving photosynthetic product formation, ultimately leading to synergistic gains in both biomass and economic yield. This establishes a solid theoretical foundation for high-yield crop cultivation. Consequently, the combined treatment of magnetized water irrigation with 25 mg/L iron proved most effective for simultaneously enhancing both *P_n_* and yield in pakchoi, representing optimal conditions for maximizing photosynthetic performance and productivity.

The present study was conducted under controlled pot conditions in a climate chamber. While this setup allowed precise regulation of environmental factors such as light, temperature, and humidity, thereby minimizing external interference, it does not fully replicate the complexity of open-field systems. Field environments present dynamic changes in temperature, light intensity, wind, and relative humidity, which affect evaporation rates, leaf surface permeability, and ultimately the efficacy of foliar iron fertilization and magnetized water irrigation. Soil heterogeneity further influences root growth, nutrient availability, and water retention. In addition, pests and diseases in the field can interact with plant nutritional status, potentially masking or amplifying treatment effects observed in a controlled setting. As such, the magnitude of the positive impacts on photosynthesis and yield reported here may be attenuated under field conditions, and their stability may be influenced by spatial and temporal variability. Therefore, our preliminary conclusions should be viewed primarily as evidence of the physiological mechanisms underlying the synergistic interaction of magnetized water irrigation and iron fertilization. Their practical application requires validation through well-designed field trials across different crops, seasons, and locations.

## 5 Conclusions

This study demonstrates that the combined application of magnetized water irrigation and foliar iron fertilization effectively improves both net photosynthetic rate and shoot fresh weight in pakchoi. In particular, magnetized water with 25 mg/L iron resulted in enhanced physiological activity and improved adaptability to light environments, significantly expanding the usable light intensity range while increasing leaf physiological performance. This optimal treatment improved *P_n_* by 15.91%–133.29% and increased yield by 38.21%–100.37%, thereby promoting photosynthetic efficiency and establishing favorable conditions for high productivity in pakchoi. These findings provide a new approach for synergistic water–fertilizer utilization in agriculture, offering theoretical support for magnetized water irrigation technology and practical guidance for precision foliar micronutrient application, with significant implications for improving vegetable yield and quality.

## Data Availability

The original contributions presented in the study are included in the article/supplementary material. Further inquiries can be directed to the corresponding authors.

## References

[B1] Anbarasan S. Ramesh S. (2022). Photosynthesis eficiency: Advances and challenges in improving crop yield. Plant Sci. Arch. 19, 21. doi: 10.5147/PSA.2022.7.3.19

[B2] Aspinwall M. J. King J. S. McKeand S. E. Domec J. C. (2011). Leaf-level gas-exchange uniformity and photosynthetic capacity among loblolly pine (Pinus taeda L.) genotypes of contrasting inherent genetic variation. Tree Physiol. 31, 78–91. doi: 10.1093/treephys/tpq107, PMID: 21389004

[B3] Bana R. S. Jat G. S. Grover M. Bamboriya S. D. Singh D. Bansal R.. (2022). Foliar nutrient supplementation with micronutrient-embedded fertilizer increases biofortification, soil biological activity and productivity of eggplant. Sci. Rep. 12, 5146. doi: 10.1038/s41598-022-09247-0, PMID: 35338233 PMC8956703

[B4] Caliskan S. Ozkaya I. Caliskan M. E. Arslan M. (2008). The effects of nitrogen and iron fertilization on growth, yield and fertilizer use efficiency of soybean in a Mediterranean-type soil. Field Crops Res. 108, 126–132. doi: 10.1016/j.fcr.2008.04.005

[B5] Coley P. D. (1983). Herbivory and defensive characteristics of tree species in a lowland tropical forest. Ecol. Monogr. 53, 209–234. doi: 10.2307/1942495

[B6] El-Desouky H. S. Islam K. R. Bergefurd B. Gao G. Harker T. Abd-El-Dayem H.. (2021). Nano iron fertilization significantly increases tomato yield by increasing plants’vegetable growth and photosynthetic efficiency. J. Plant Nutr. 44, 1649–1663. doi: 10.1080/01904167.2021.1871749

[B7] Gao D. Ran C. Zhang Y. Wang X. Lu S. Geng Y.. (2022). Effect of different concentrations of foliar iron fertilizer on chlorophyll fluorescence characteristics of iron-deficient rice seedlings under saline sodic conditions. Plant Physiol. Biochem. 185, 112–122. doi: 10.1016/j.plaphy.2022.05.021, PMID: 35671588

[B8] Guiamba H. D. S. S. Zhang X. Sierka E. Lin K. Ali M. M. Ali W. M.. (2022). Enhancement of photosynthesis efficiency and yield of strawberry (Fragaria ananassa Duch.) plants via LED systems. Front. Plant Sci. 13. doi: 10.3389/fpls.2022.918038, PMID: 36161001 PMC9507429

[B9] Guo Y. Lv Y. (2025). Evaluation of models for describing photosynthetic light–response curves and estimating parameters in rice leaves at various canopy positions. Agronomy 15, 125. doi: 10.3390/agronomy15010125

[B10] Hu W. Lu Z. Meng F. Li X. Cong R. Ren T.. (2020). The reduction in leaf area precedes that in photosynthesis under potassium deficiency: the importance of leaf anatomy. New Phytol. 227, 1749–1763. doi: 10.1111/nph.16644, PMID: 32367581

[B11] Hussein A. S. Abeed A. H. Usman A. R. Abou-Zaid E. A. (2024). Conventional vs. nano-micronutrients as foliar fertilization for enhancing the quality and nutritional status of pomegranate fruits. J. Saudi Soc. Agric. Sci. 23, 112–122. doi: 10.1016/j.jssas.2023.09.008

[B12] Ishfaq M. Kiran A. ur Rehman H. Farooq M. Ijaz N. H. Nadeem F.. (2022). Foliar nutrition: Potential and challenges under multifaceted agriculture. Environ. Exp. Bot. 200, 104909. doi: 10.1016/j.envexpbot.2022.104909

[B13] Januszkiewicz R. Kulczycki G. Samoraj M. (2023). Foliar fertilization of crop plants in polish agriculture. Agriculture 13, 1715. doi: 10.3390/agriculture13091715

[B14] Kieffer C. Kaur N. Li J. Matamala R. Fay P. A. Hui D. (2024). Photosynthetic responses of switchgrass to light and CO_2_ under different precipitation treatments. GCB Bioenergy 16, e13138. doi: 10.1111/gcbb.13138

[B15] Kobayashi T. Nishizawa N. K. (2012). Iron uptake, translocation, and regulation in higher plants. Annu. Rev. Plant Biol. 63, 131–152. doi: 10.1146/annurev-arplant-042811-105522, PMID: 22404471

[B16] Kolbert Z. Cuypers A. Verbruggen N. (2022). Essential trace metals: micronutrients with large impact. J. Exp. Bot. 73, 1685–1687. doi: 10.1093/jxb/erac025, PMID: 35288752

[B17] Ma L. Li Y. Wu P. Zhao X. Gao X. Chen X. (2020). Recovery growth and water use of intercropped maize following wheat harvest in wheat/maize relay strip intercropping. Field Crops Res. 256, 107924. doi: 10.1016/j.fcr.2020.107924

[B18] Maheshwari B. L. Grewal H. S. (2009). Magnetic treatment of irrigation water: Its effects on vegetable crop yield and water productivity. Agric. Water Manage. 96, 1229–1236. doi: 10.1016/j.agwat.2009.03.016

[B19] Merry R. Dobbels A. A. Sadok W. Naeve S. Stupar R. M. Lorenz A. J. (2022). Iron deficiency in soybean. Crop Sci. 62, 36–52. doi: 10.1002/csc2.20661

[B20] Mu Y. Zhao G. Q. Zhao Q. Q. Liu H. Wang Q. J. (2019). Advances in the application of activated water irrigation. J. Agric. Resour. Environ. 36, 403–411. doi: 10.13254/j.jare.2019.0106

[B21] Nasar J. Wang G. Y. Zhou F. J. Gitari H. Zhou X. B. Tabl K. M.. (2022). Nitrogen fertilization coupled with foliar application of iron and molybdenum improves shade tolerance of soybean under maize-soybean intercropping. Front. Plant Sci. 13. doi: 10.3389/fpls.2022.1014640, PMID: 36267939 PMC9577300

[B22] Niu Y. Lyu H. Liu X. Zhang M. Li H. (2023). Photosynthesis prediction and light spectra optimization of greenhouse tomato based on response of red–blue ratio. Scientia Hortic. 318, 112065. doi: 10.1016/j.scienta.2023.112065

[B23] Nyakane N. E. Markus E. D. Sedibe M. M. (2019). The effects of magnetic fields on plants growth: a comprehensive review. Int. J. Food Eng. 5, 79–87. doi: 10.18178/ijfe.5.1.79-87

[B24] Okla M. K. Saleem M. H. Saleh I. A. Zomot N. Perveen S. Parveen A.. (2023). Foliar application of iron-lysine to boost growth attributes, photosynthetic pigments and biochemical defense system in canola (Brassica napus L.) under cadmium stress. BMC Plant Biol. 23, 648. doi: 10.1186/s12870-023-04672-3, PMID: 38102555 PMC10724993

[B25] Putti F. F. Vicente E. F. Chaves P. P. N. Mantoan L. P. B. Cremasco C. P. Arruda B.. (2023). Effect of magnetic water treatment on the growth, nutritional status, and yield of lettuce plants with irrigation rate. Horticulturae 9, 504. doi: 10.3390/horticulturae9040504

[B26] Qu S. Lv J. Liu J. (2020). Visualization analysis for global water resources based on digital earth. J. Coast. Res. 105, 47–50. doi: 10.2112/JCR-SI105-010.1

[B27] Quanjiu W. Yan S. Songrui N. Jihong Z. Beibei Z. Lijun S.. (2019). Effects of activated irrigation water on soil physicochemical properties and crop growth and analysis of the probable pathway. Adv. Earth Sci. 34, 660–670.

[B28] Shabrangy A. Ahmad M. (2009). Effect of magnetic fields on growth and antioxidant systems in agricultural plants PIERS proceedings (Beijing, China: PIERS Proceedings), 23–27.

[B29] Simkin A. J. López-Calcagno P. E. Raines C. A. (2019). Feeding the world: improving photosynthetic efficiency for sustainable crop production. J. Exp. Bot. 70, 1119–1140. doi: 10.1093/jxb/ery445, PMID: 30772919 PMC6395887

[B30] Sun S. Feng Y. Huang G. Zhao X. Song F. (2022). Rhizophagus irregularis enhances tolerance to cadmium stress by altering host plant hemp (Cannabis sativa L.) photosynthetic properties. Environ. pollut. 314, 120309. doi: 10.1016/j.envpol.2022.120309, PMID: 36181931

[B31] Sun Y. Wang J. Wang Q. Wang C. (2023). Responses of the growth characteristics of spinach to different moisture contents in soil under irrigation with magnetoelectric water. Agronomy 13, 657. doi: 10.3390/agronomy13030657

[B32] Sun Y. Wang C. Wang Q. Wang J. Wang Y. Li M.. (2024). Effects of magnetoelectric water irrigation combined with foliar iron fertilizer on the growth characteristics and iron absorption of spinach. Scientia Hortic. 327, 112824. doi: 10.1016/j.scienta.2023.112824

[B33] Thornley J. H. (1976). Mathematical models in plant physiology. (Pittsburgh: Academic Press), 318.

[B34] Wan L. Xing Z. Chang X. Liu J. Zhang G. (2018). Research on light response curve fitting model of four Chamaenerion plants on the Serzilla Mountains. Am. J. Plant Sci. 9, 1630. doi: 10.4236/ajps.2018.98118

[B35] Wang T. Cheng K. Huo X. Meng P. Cai Z. Wang Z.. (2022). Bioorganic fertilizer promotes pakchoi growth and shapes the soil microbial structure. Front. Plant Sci. 13. doi: 10.3389/fpls.2022.1040437, PMID: 36426155 PMC9679507

[B36] Wang Y. Kang Y. Zhong M. Zhang L. Chai X. Jiang X.. (2022). Effects of iron deficiency stress on plant growth and quality in flowering Chinese cabbage and its adaptive response. Agronomy 12, 875. doi: 10.3390/agronomy12040875

[B37] Wei K. Zhang J. Wang Q. Chen Y. Ding Q. (2021). Effects of ionized brackish water and polyacrylamide application on infiltration characteristics and improving water retention and reducing soil salinity. Can. J. Soil Sci. 101, 324–334. doi: 10.1139/cjss-2020-0099

[B38] Xing W. Huang W. Liu G. (2010). Effect of excess iron and copper on physiology of aquatic plant Spirodela polyrrhiza (L.) Schleid. Environ. Toxicology: Int. J. 25, 103–112. doi: 10.1002/tox.20480, PMID: 19260045

[B39] Yamori N. Levine C. P. Mattson N. S. Yamori W. (2022). Optimum root zone temperature of photosynthesis and plant growth depends on air temperature in lettuce plants. Plant Mol. Biol. 110, 385–395. doi: 10.1007/s11103-022-01249-w, PMID: 35169910

[B40] Ye Z. P. (2007). A new model for relationship between irradiance and the rate of photosynthesis in Oryza sativa. Photosynthetica 45, 637–640. doi: 10.1007/s11099-007-0110-5

[B41] Ye Z. P. Suggett D. J. Robakowski P. Kang H. J. (2013). A mechanistic model for the photosynthesis–light response based on the photosynthetic electron transport of photosystem II in C3 and C4 species. New Phytol. 199, 110–120. doi: 10.1111/nph.12242, PMID: 23521402

[B42] Zhang S. Gong J. Xiao C. Yang X. Li X. Zhang Z.. (2024). Bupleurum chinense and Medicago sativa sustain their growth in agrophotovoltaic systems by regulating photosynthetic mechanisms. Renewable Sustain. Energy Rev. 189, 114024. doi: 10.1016/j.rser.2023.114024

[B43] Zhang Y. Liang Y. Han J. Hu X. Li X. Zhao H.. (2023). Interactive effects of iron and photoperiods on tomato plant growth and fruit quality. J. Plant Growth Regul. 42, 376–389. doi: 10.1007/s00344-021-10554-5

[B44] Zhang J. Sun B. Yang C. Wang C. You Y. Zhou G.. (2022). A novel composite vegetation index including solar-induced chlorophyll fluorescence for seedling rapeseed net photosynthesis rate retrieval. Comput. Electron. Agric. 198, 107031. doi: 10.1016/j.compag.2022.107031

[B45] Zhang R. Zhang W. Kang Y. Shi M. Yang X. Li H.. (2022). Application of different foliar iron fertilizers for improving the photosynthesis and tuber quality of potato (Solanum tuberosum L.) and enhancing iron biofortification. Chem. Biol. Technol. Agric. 9, 79. doi: 10.1186/s40538-022-00346-8

[B46] Zhao G. Mu Y. Wang Y. Wang L. (2022). Magnetization and oxidation of irrigation water to improve winter wheat (Triticum aestivum L.) production and water-use efficiency. Agric. Water Manage. 259, 107254. doi: 10.1016/j.agwat.2021.107254

[B47] Zhao G. Zhou B. Mu Y. Wang Y. Liu Y. Wang L. (2021). Irrigation with activated water promotes root growth and improves water use of winter wheat. Agronomy 11, 2459. doi: 10.3390/agronomy11122459

[B48] Zheng M. Sun Y. Wang Q. Bai Y. Mu W. Zhang J.. (2024). Coupled efficacy of magneto-electric water irrigation with foliar iron fertilization for spinach growth. Agronomy 14, 1482. doi: 10.3390/agronomy14071482

[B49] Zhou J. Li P. Wang J. (2022). Effects of light intensity and temperature on the photosynthesis characteristics and yield of lettuce. Horticulturae 8, 178. doi: 10.3390/horticulturae8020178

